# The Value of Diffusion-Weighted Imaging in the Differential Diagnosis of Ovarian Lesions: A Meta-Analysis

**DOI:** 10.1371/journal.pone.0149465

**Published:** 2016-02-23

**Authors:** Hyun-Jung Kim, So-Yeon Lee, Yu Ri Shin, Chang Suk Park, Kijun Kim

**Affiliations:** 1 Department of Preventive Medicine, College of Medicine, Korea University, Seoul, Korea; 2 Department of Radiology, Kangbuk Samsung Hospital, Sungkyunkwan University School of Medicine, Seoul, Republic of Korea; 3 Department of Radiology, College of Medicine, Incheon St. Mary's Hospital, The Catholic University of Korea, Incheon, Republic of Korea; Suzhou University, CHINA

## Abstract

**Objectives:**

The ability of contrast-enhanced MRI to distinguish between malignant and benign ovarian masses is limited. The aim of this meta-analysis is to evaluate the diagnostic performance of diffusion-weighted imaging (DWI) in differentiating malignant from benign ovarian masses.

**Methods:**

A comprehensive literature search was performed in several authoritative databases to identify relevant articles. The weighted mean difference (WMD) and corresponding 95% confidence interval (95% CI) were calculated. We also used subgroup analysis to analyze study heterogeneity, and evaluated publication bias.

**Results:**

The meta-analysis is based on 21 studies, which reported the findings for 731 malignant and 918 benign ovarian masses. There was no significant difference in apparent diffusion coefficient (ADC) values for DWI between benign and malignant lesions (WMD = 0.22, 95% CI = -0.02–0.47, p = 0.08). Subgroup analysis by benign tumor type revealed higher ADC values (or a trend toward higher values) for cysts, cystadenomas and other benign tumors compared to malignant masses (cyst: WMD = 0.54, 95% CI = -0.05–1.12, p = 0.07; cystadenoma: WMD = 0.73, 95% CI = 0.38–1.07, p < 0.0001; other benign tumor: WMD = 0.16, 95% CI = -0.13–0.46, p = 0.28). On the other hand, lower ADC values (or a trend toward lower values) were observed for endometrioma and teratoma compared to malignant masses (endometrioma: WMD = -0.09, 95% CI = -0.47–0.29, p = 0.64; teratoma: WMD = -0.49, 95% CI = -0.85–0.12, p = 0.009). Subgroup analysis by mass property revealed higher ADC values in cystic tumor types than in solid types for both benign and malignant tumors. Significant study heterogeneity was observed. There was no notable publication bias.

**Conclusions:**

Quantitative DWI is not a reliable diagnostic method for differentiation between benign and malignant ovarian masses. This knowledge is essential in avoiding misdiagnosis of ovarian masses.

## Introduction

Ovarian cancer is a leading cause of death among gynecological malignancies, and the fifth most common cause of cancer deaths in women [1]. Because most patients with ovarian cancer present in an advanced stage of the disease due to its silent clinical course, a non-invasive and accurate diagnostic tool would be highly desirable. The main goal of imaging techniques in this setting is to differentiate malignant tumors from benign ones, and to determine the surgical strategy. Magnetic resonance imaging (MRI) is a highly accurate technique for detection and characterization of ovarian masses, with gadolinium-enhanced MRI studies providing the best assessment of complex ovarian masses. However, despite the use of these excellent imaging techniques—which have superb spatial and contrast resolution—radiologists still have difficulty determining the malignant potential of ovarian masses. These limitations have spurred the desire for other useful MRI techniques, such as diffusion-weighted imaging (DWI). This modality enables the radiologist to move from morphological to functional assessment of disease by providing information about tissue consistency and the integrity of cell membranes; moreover, it permits quantitative assessment via the measurement of apparent diffusion coefficient (ADC) values [2]. In addition, patients with renal insufficiency who undergo contrast-enhanced MRI have an increased risk of developing nephrogenic systemic fibrosis; [3] thus, there is a clinical need for non-enhanced imaging modalities that might be useful for preoperative evaluation of ovarian masses.

DWI sequences characterize the restriction of random (Brownian) movement of water molecules within tissues. The strength of diffusion weighting is characterized by the diffusion gradient factor, or ‘*b* value’. Through linear regression, images taken at various *b* values can provide quantitative measurement of ADC values in a region of interest [4]. Because ADC is related to the molecular translational movement of water molecules, increased tissue cellularity or cell density decreases ADC value[5]. In general, malignant tumors have hyper-cellularity, enlarged nuclei, and angulation of nuclear contour than benign lesions; therefore, ADC values assist in distinguishing between benign and malignant lesions [6, 7]. Although multiple studies have examined the utility of DWI in the differential diagnosis of malignant from benign tumors, findings have been largely incongruent; some studies have shown that DWI is helpful in distinguishing benign and malignant ovarian masses [8–10], while others have presented inconsistent results [11–13]. We therefore conducted this meta-analysis to further evaluate the utility of DWI for discrimination of benign and malignant ovarian masses, and to determine the diagnostic accuracy of ADC values for ovarian mass characterization.

## Materials and Methods

Our systematic review and meta-analysis was performed according to the Cochrane Review Methods [14]. Results were reported in accordance with the PRISMA (Preferred Reporting Items for Systematic Reviews and Meta-Analyses) statement (S1 PRISMA Checklist) [15].

### Data Sources & Literature Search

A literature search of the MEDLINE, EMBASE, and Cochrane Central Register of Controlled Trials (CENTRAL) databases through June 30, 2015 was conducted using the following keywords and subject headings: (“DWI” or “diffusion weighted imaging” or “diffusion weighted MRI”) and (“ovary” or “ovarian cancer” or “neoplasm” or “tumor” or “ovarian cyst” or “malignant” or “benign”) (see S1 Table for the comprehensive list). The search was restricted to human subjects, with no restrictions on language or year of publication. After the initial electronic search, additional relevant articles were identified by conducting a hand search of references from the identified studies.

### Study Selection

Studies were chosen independently by two authors (YRS and SYL) based on the selection criteria. A multi-level screening process was used: at the first level, we reviewed the title and abstract of each paper; at the second level, we reviewed the full text of potentially relevant references. Studies were included in the meta-analysis if: (1) the authors attempted to determine the benignity or malignancy of ovarian masses; (2) data were obtained by calculation of ADC values using either 1.5T or 3.0T MRI; (3) sufficient data were provided to complete the fields of a constructed table; and (4) histopathology results and/or clinical follow-up were used as the reference standard. The exclusion criteria were as follows: (1) case reports and case series with a sample size of fewer than 8 patients; (2) review articles, editorials, letters, abstracts, and comments; (3) studies not within the field of interest of this study; (4) studies with insufficient data to complete the constructed table; (5) studies in which the relevant data overlapped with that of other studies due to patient overlap; and (6) studies of borderline ovarian tumors, which have distinct characteristics compared to benign or malignant ovarian masses.

### Data Extraction

The two authors (YRS and SYL) independently extracted data from each study using a predefined data extraction form. All discrepancies in data extraction were resolved by discussion with the third author (HJK).

The following variables were extracted from studies: (1) patient baseline characteristics (source country, patient age, number of masses, type of ovarian tumor (benign, including cysts, cystadenoma, endometrioma, teratoma, and other benign tumors; or malignant, including epithelial and non-epithelial)); (2) study design (prospectively or retrospective); (3) choice of reference standard; (4) image protocols used for DWI (MRI machine type, magnetic field strength, slice thickness of images, *b* values, and diagnostic threshold); and (5) diagnostic value (mean and standard deviation (SD) of ADC in benign and malignant tumors, sensitivity and specificity). For subgroup analysis, benign tumors were further categorized as cysts, cystadenoma (serous and mucinous), endometrioma, teratoma, or other benign tumor (fibroma, thecoma, adenofibroma, etc.). Malignant tumors were further categorized as epithelial or non-epithelial (sex-cord or germ cell) tumors. Tumors were also categorized by mass property (solid or cystic).

### Assessment of Methodological Quality

Two authors (YRS and SYL) independently assessed the methodological qualities of each study using the QUADAS-2 (Quality Assessment of Diagnostic Accuracy Studies) tool [16]. Any disagreements were resolved through discussion or input from the third author (HJK).

### Statistical Analysis

The weighted mean differences (WMD) for the ADC values in benign and malignant ovarian masses were calculated. A 95% confidence interval (95% CI) was calculated for the summary WMD using a Z test. Diagnostic performance was summarized using a random-effects coefficient binary regression model [17]. In addition, a test for heterogeneity between the trials included for each comparison was performed using the *I*^2^ statistic, with *I*^2^ > 50% indicating the presence of heterogeneity [18]. If notable statistical or clinical heterogeneity was observed, we planned to investigate the possible causes using pre-specified subgroup analyses. Subgroup analyses were carried out on different lesion classes and types. Egger’s linear regression test and visual inspection of the funnel plot were applied to detect potential publication bias [19, 20]. We used RevMan version 5.2 (The Cochrane Collaboration, Oxford, UK) and STATA version 13.0 (Stata Corporation, College Station, TX, USA) for these analyses.

## Results

### Identification of Studies

**Fig 1:**
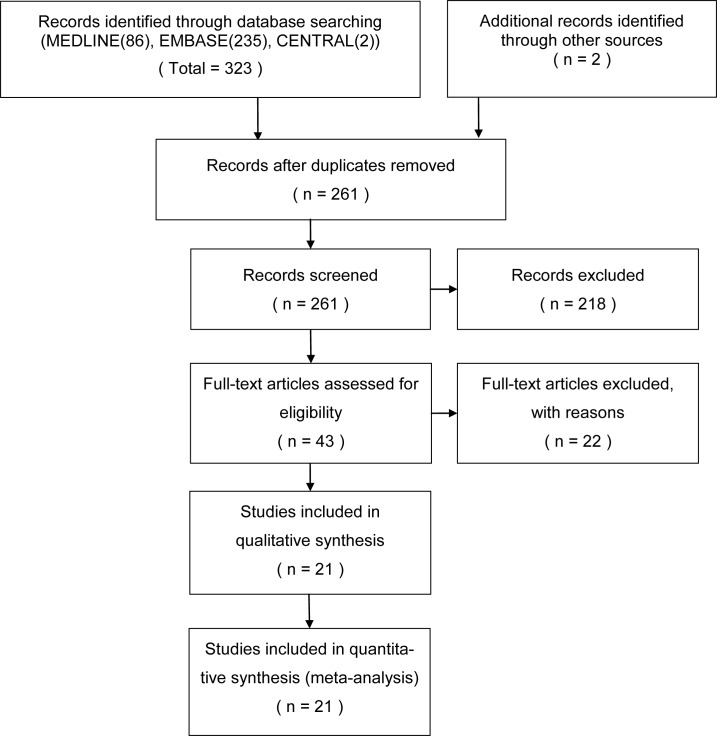
Flow chart describing the study selection process. From: www.prisma-statement.org. Used with permission.

The systematic database search returned 261 articles. Of these, 218 were excluded as their titles and abstracts indicated that they did not fulfill the selection criteria. Full manuscripts were obtained for the remaining 43 articles; following scrutiny of these, 22 publications were excluded as they were not cohort studies (n = 1), were not in the field of interest (n = 5), were not relevant to DWI (n = 2), or had insufficient data (n = 13) or patient overlap (n = 1). Therefore, a total of 21 studies were included in the review [8–13, 21–35]. The study selection process is presented with a flow chart in Fig 1.

### Study Characteristics and Quality Assessment

Table 1 shows the principal characteristics of the included studies. In total, 21 studies (3 prospective, 18 retrospective) comprising 731 malignant ovarian masses and 918 benign ovarian masses were incorporated into the meta-analysis. MRI machine types included GE 1.5/3.0T scanners, Siemens 1.5T scanners, and Philips 3.0T scanners. ADC values (x 10^−3^ mm^2^/s) were expressed as mean ±SD, and ADC levels in malignant and benign ovarian masses were recorded. The results of quality assessment are shown in S2 Table. The overall methodological quality of the included studies was fair. The risk of bias related to patient selection, the reference standard, and flow and timing was low in all studies; however, there was significant risk of bias related to the index test, due to considerable heterogeneity.

**Table 1:** The Principal Characteristics of 21 Eligible Studies Included in the Meta-Analysis.

Author	Year	Country	Design	MRI-machine-type	*b* value	Reference standard	No. of benign masses	No. of malignant masses	Threshold of ADC (x10^-3^mm^2^/s)
Bakir et al. [12]	2011	Turkey	retrospective	Siemens 1.5T	50, 400, 800	histopathology	2	18	ND
Cappabianca et al. [21]	2013	Italy	retrospective	Siemens 1.5T	0, 500, 1000	histopathology	72	60	ND
Chilla et al. [22]	2011	Switzerland	prospective	Siemens 1.5T	50, 400, 800	histopathology	30	6	ND
Fujii et al. [11]	2008	Japan	retrospective	Siemens 1.5T	0, 1000	histopathology	81	42	ND
Heo et al. [23]	2005	Korea	retrospective	GE 1.5T	0, 800	Histopathology, FU imaging	19	7	ND
Inci et al. [24]	2011	Turkey	retrospective	Siemens 1.5T	0, 500, 1000	Histopathology, FU imaging	48	11	ND
Katayama et al. [13]	2002	Japan	retrospective	GE 1.5T	200, 400, 600	histopathology	56	10	ND
Kierans et al. [25]	2013	USA	retrospective	Siemens 1.5T	0, 500	histopathology	30	9	ND
Koc et al. [26]	2012	Turkey	retrospective	Siemens 1.5T	0, 50, 200, 400, 500, 600, 800, 1000	histopathology	20	11	1.2
Kozawa et al. [27]	2014	Japan	retrospective	Siemens 1.5T, Philips 3.0T	0, 500, 1000	histopathology	11	5	ND
Li et al. [9]	2012	China	retrospective	GE 1.5T	0, 1000	histopathology	54	124	1.25
Moteki et al. [28]	2000	Japan	retrospective	GE 1.5T	0, 200	Histopathology, FU imaging	49	12	ND
Nakayama et al. [10]	2005	Japan	retrospective	Siemens 1.5T	0, 500, 1000	histopathology	99	24	ND
Takeuchi et al. [29]	2010	Japan	retrospective	GE 1.5T/3.0T	0, 800	histopathology	10	39	1.15 / 1
Takeuchi et al. [30]	2011	Japan	retrospective	GE 1.5T/3.0T	0, 800	histopathology	11	18	ND
Takeuchi et al. [31]	2013	Japan	retrospective	GE 1.5T/3.0T	0, 800	histopathology	11	27	1.2
Thomassin et al. [8]	2009	France	prospective	Siemens 1.5T	0, 500, 1000	histopathology	33	81	ND
Zhang et al. [32]	2012	China	retrospective	GE 1.5T	0, 1000	histopathology	74	128	1.2
Zhang et al. [33]	2012	China	retrospective	GE 3.0T	0, 700	histopathology	98	42	ND
Zhang et al. [34]	2013	China	retrospective	GE 3.0T	0, 700	histopathology	23	19	ND
Zhang et al. [35]	2014	China	prospective	GE 3.0T	0, 700	histopathology	87	38	ND

FU, follow-up; ND, not documented

### Diagnostic Performance

**Fig 2:**
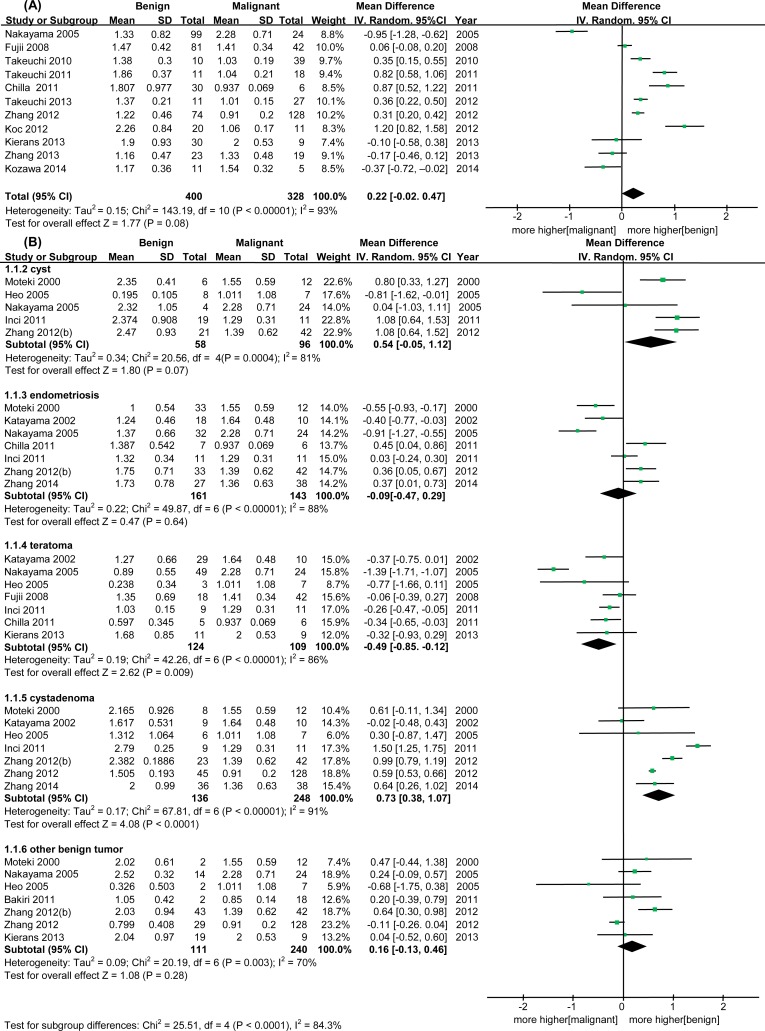
Forest plot for the diagnostic significance of diffusion-weighted MRI between benign and malignant ovarian tumors; apparent diffusion coefficient (ADC) values. (A) Overall, (B) Subgroup analysis according to benign tumor type.

**Fig 3:**
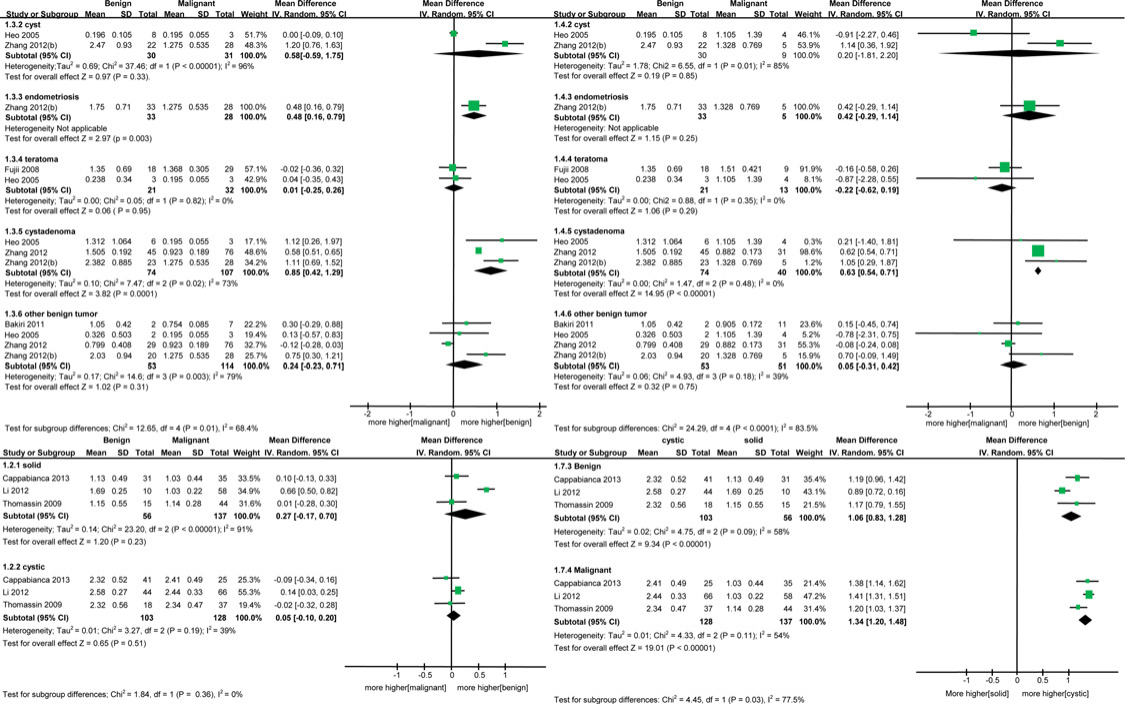
Forest plots of subgroup analyses according to malignant tumor type and tumor property. (A) Epithelial malignant tumors, (B) Non-epithelial malignant tumors, (C and D) Solid and cystic tumors.

The meta-analysis revealed no statistically significant differences in ADC values between benign and malignant ovarian masses (WMD = 0.22, 95% CI = -0.02–0.47, *I*^2^ = 93%, p = 0.08) (Fig 2A). When studies were stratified by tumor type to explore potential sources of heterogeneity, ADC values were found to be higher in cystadenomas than in malignant masses (WMD = 0.73, 95% CI = 0.38–1.07, p < 0.0001). There was a trend toward higher ADC values in cysts and other benign tumors compared to malignant tumors, although this difference was not statistically significant (cysts: WMD = 0.54, 95% CI = -0.05–1.12, p = 0.07; other benign tumors: WMD = 0.16, 95% CI = -0.13–0.46, p = 0.28). On the other hand, teratomas had lower ADC values in comparison to malignant masses (WMD = -0.49, 95% CI = -0.85–0.12, p = 0.009), and there was also a non-significant trend toward lower ADC values in endometrioma (WMD = -0.09, 95% CI = -0.47–0.29, p = 0.64) (Fig 2B). Subgroup analysis by epithelial and non-epithelial malignant tumor types revealed that differences in ADC values between benign and malignant ovarian masses are greater in epithelial malignant tumor subgroups (Fig 3A and 3B). The reported ranges of ADC values in benign and malignant ovarian tumors are given in S1 Fig.

Studies were also stratified by solid or cystic tumor properties; no significant difference in ADC values was found between benign and malignant masses in these subgroups (solid: WMD = 0.27, 95% CI = -0.17–0.7, p = 0.23; cystic: WMD = 0.05, 95% CI = -0.1–0.2, p = 0.51) (Fig 3C). Higher ADC values in cystic groups compared to solid groups were observed in both benign and malignant tumors (benign: WMD = 1.06, 95% CI = 0.83–1.28, p < 0.00001; malignant: WMD = 1.34, 95% CI = 1.2–1.48, p < 0.00001) (Fig 3D).

**Fig 4:**
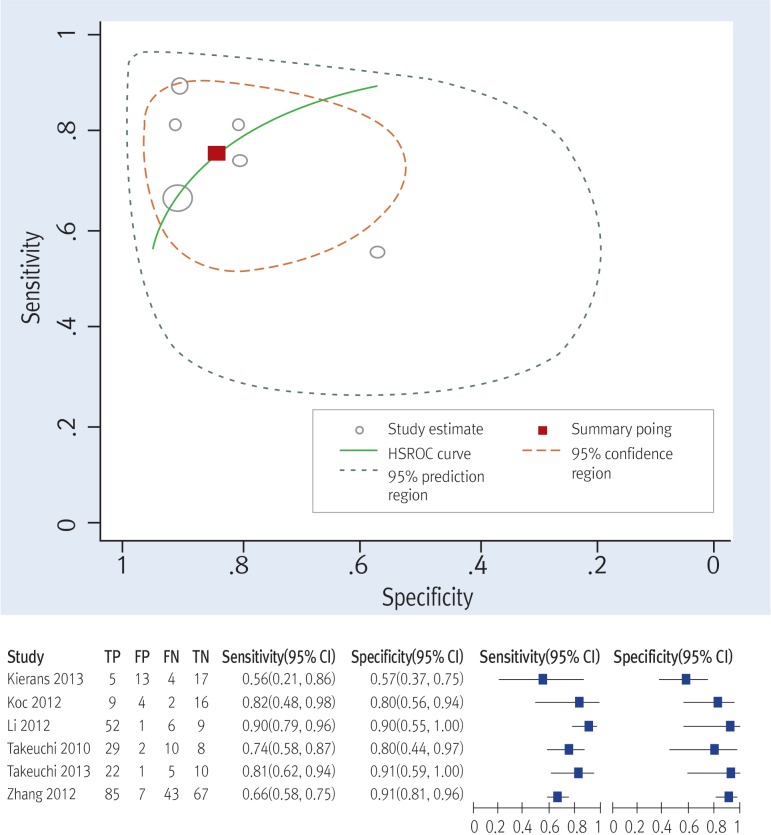
Hierarchical summary receiver operating characteristic (HSROC) curves of diffusion-weighted MRI and coupled forest plot.

A hierarchical summary receiver-operator curve (HSROC) with a 95% confidence ellipse (Fig 4) presents a global summary of test performance, and yielded a maximum joint sensitivity of 0.76 (95% CI = 0.64–0.85) and specificity of 0.84 (95% CI = 0.69–0.92), indicating a low level of overall accuracy.

### Publication Bias

Egger’s regression test (p = 0.774) showed that studies were distributed symmetrically in the funnel plot of mean ADC values for benign and malignant ovarian masses, indicating that publication bias was not detected in this systematic review (S2 Fig).

## Discussion

Studies of DWI to date have reported various levels of diagnostic accuracy for distinguishing between benign and malignant ovarian masses. Li et al. [9] reported that the mean ADC value of malignant ovarian masses was significantly lower than that of benign ovarian masses, and that ADC could therefore be used to differentiate between the two. In contrast, Fujii et al. [11], Bakir et al. [12] and Katayama et al. [13] have each suggested that DWI is not effective for differentiating malignant from benign ovarian masses. A meta-analysis may resolve these incongruities by increasing sample size, reducing random error, increasing testing efficiency, and improving accuracy of evaluation of the effect size. The present meta-analysis identified 21 studies that examined the diagnostic accuracy of DWI for characterization of ovarian masses. To our knowledge, this is the first meta-analysis to use a complex statistical analysis to evaluate the role of DWI in ovarian mass characterization.

Our meta-analysis revealed no statistically significant difference in ADC values between benign and malignant ovarian masses, implying that DWI may not be effective in differentiating malignant ovarian tumors from benign. The poor performance of DWI for this application may be due to the abnormal signal intensity of DWI in benign lesions, especially teratoma and endometrioma. These types of lesions exhibit very low ADC values, overlapping with those typical of malignant tumors and resulting in reduced diagnostic accuracy. The low ADC value of teratoma has been attributed to the keratinoid content of this type of tumor [10]. Nearly half of endometriomas have also been found to demonstrate abnormal signal intensity on DWI, due to high concentrations of blood and hemosiderin [13]. Hemosiderin contains iron, a strong paramagnetic substance, which could reduce the T1 value and decrease the ADC [36]. However, both teratomas and endometriomas are in most cases easily identified using conventional MRI sequences, without the need for integration with DWI. When endometriomas and teratomas exhibiting low ADC values have been excluded by use of conventional MRI sequences, ADC mapping may be helpful for detection of malignancy in mixed solid and cystic masses. Exclusion of endometriomas and teratomas could be considered a potential bias; however, as recommended by Moteki et al., all tumors displaying a high T1 signal before the DWI sequence should be excluded to limit T1 contamination, because ADC values may increase linearly with decreasing protein concentration [37].

There was some overlap between the mean ADC values of malignant and benign ovarian lesions. This result may reflect the increased mean ADC values of some malignant lesions (due to the existence of small necrotic or cystic areas in solid tumoral components, desmoplastic reaction in the stroma, or fluid collection in intervening papillary components) in conjunction with the decreased mean ADC values of some benign lesions (due to the presence of abundant collagen-producing fibroblastic cells and a dense network of collagen fibers in the extracellular matrix). To avoid these pitfalls, diffusion-weighted images and ADC maps should be interpreted together with other images, such as T2-weighted images and enhancement characteristics on T1-weighted images.

ADC values were higher in cystic lesions compared to solid lesions for both benign and malignant ovarian masses. We consider these results reasonable, since ADC differences could be caused mainly by the tissue’s structure and its components and reflects tissue viscosity [38]. ADC is inversely correlated with the protein content of the cystic component, which is elevated by the presence of hemorrhagic and mucinous material; [28, 37] thus, ADC values are more closely related to the density of a mass rather than to a particular histopathologic group.

Mass size is also a potential source of heterogeneity, although we were not able to perform a subgroup analysis for this characteristic due to limited data. Huge cystic masses (larger than 12 cm) are subject to the ‘sloshing effect,’ in which intermittent compression of the mass by abdominal wall movement during breathing induces intracystic turbulent flow, resulting in marked signal loss on DWI [28]. This phenomenon may affect ADC values for DWI acquired through free-breathing imaging. The sloshing effect is the dominant cause of elevated ADC values for huge cystic ovarian masses, independent of their content; thus, ADC analysis may be more suitable for small to medium cystic ovarian masses [28].

We should acknowledge some limitations in this meta-analysis. First, considerable heterogeneity was identified among the included studies, and it was necessary to explore the reasons for this. The results of ADC calculation may be affected by the parameters used in DWI sequences, *b* value being one of the most important [39]. There is currently no standardized DWI scanning method or acquisition protocol; thus, differences in MRI scanner vendors, magnetic field strength and sequences, and choice of *b* values may all contribute to between-study heterogeneity. In the low *b*-value range (less than 200 s/mm^2^), signals are significantly affected by perfusion effects, leading to inaccurate reflection of water diffusion motion [40]. Conversely, high *b* values carry the risk of distortion and susceptibility artifacts [41]. Cross-study variability in *b* values makes the ranges and thresholds of ADC values difficult to interpret. Intravoxel incoherent motion (IVIM) can also contribute heavily to increased variability in ADC measurement across studies, and calculation of ADC should be biexponential, rather than monoexponential as has been commonly performed to date [42]. All of the above factors can affect ADC measurements and may in part explain the observed variability in ADC cut-off values, which ranged from 0.778 to 1.150 x 10^−3^ mm^2^/s; in addition, the ADC value threshold is likely machine-specific and not universal. Finally, most of the included studies were retrospective in design, which could affect the diagnostic performance of DWI and may be considered a limitation.

In conclusion, quantitative DWI is not a reliable diagnostic method for differentiation between benign and malignant ovarian masses. Because ADC values may vary by benign tumor type, results must be interpreted with caution. Although DWI imaging may have some utility in characterizing ovarian masses, large prospective studies with stricter standardization of DWI protocols and integral data are necessary to determine a routine clinical application for this approach.

## Supporting Information

S1 PRISMA Checklist PRISMA Checklist. (DOC)

S1 Fig Ranges of ADC values in benign and malignant ovarian tumors. (TIF)

S2 Fig Funnel plots for visual assessment of publication bias. (TIF)

S1 Table Search Strategy. (DOC)

S2 Table Methodological Quality of 21 Included Studies Using the QUADAS-2 tool. (DOC)
