# Supplementary figures and images for: The Value of Diffusion-Weighted Imaging in the Differential Diagnosis of Ovarian Lesions: A Meta-Analysis
Source: PLoS One. 2016 Feb 23;11(2):e0149465. doi: 10.1371/journal.pone.0149465 (PMC4764370; doi:10.1371/journal.pone.0149465)

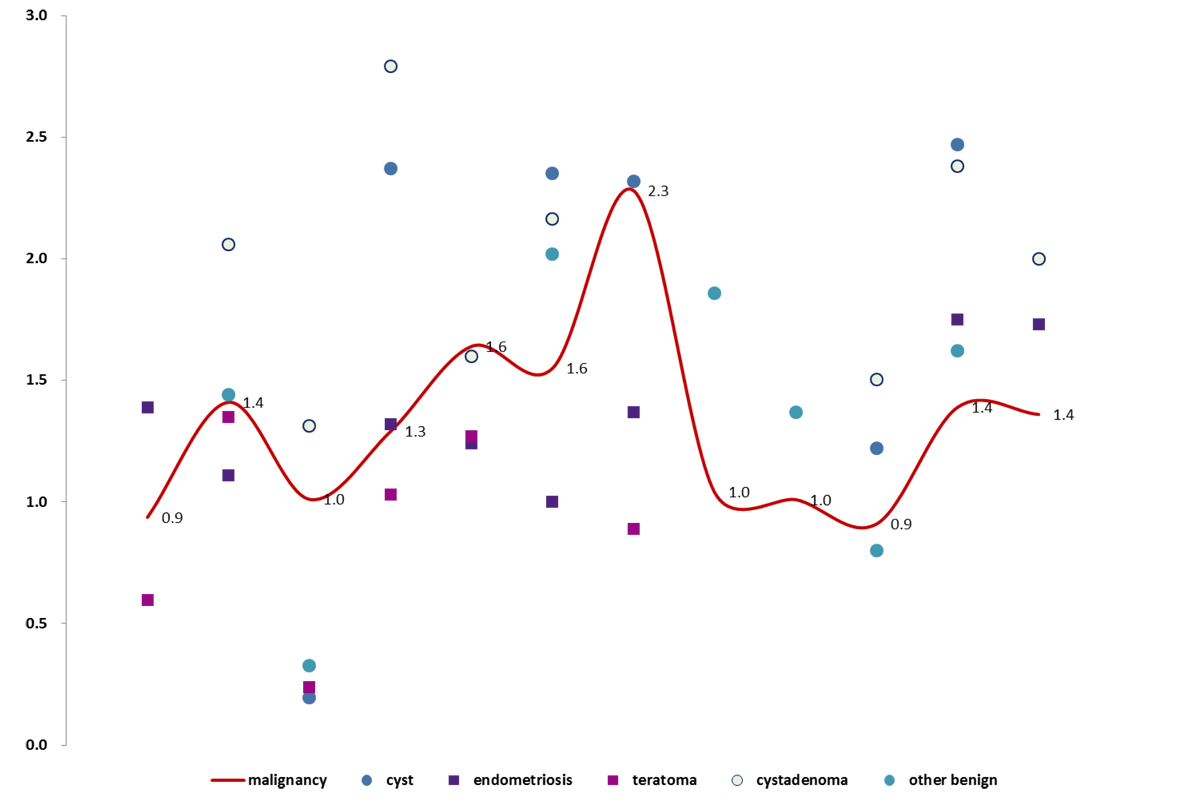

Supplement: S1 Fig — (TIF) [file pone.0149465.s002.tif]

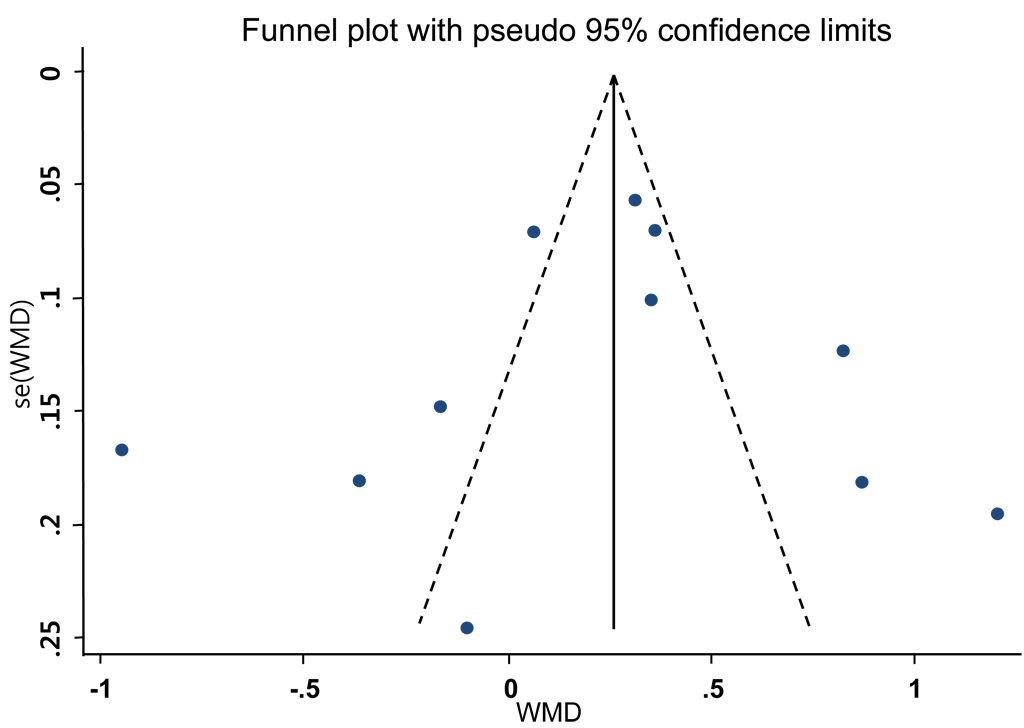

Supplement: S2 Fig — (TIF) [file pone.0149465.s003.tif]
