# Supplementary material for: The Value of Diffusion-Weighted Imaging in the Differential Diagnosis of Ovarian Lesions: A Meta-Analysis
Source: PLoS One. 2016 Feb 23;11(2):e0149465. doi: 10.1371/journal.pone.0149465 (PMC4764370; doi:10.1371/journal.pone.0149465)
Supplement: S1 Table — (DOC) [file pone.0149465.s004.doc]

| Database | Search strategy |
| --- | --- |
| MEDLINE | 1. "Ovarian Neoplasms"[tiab] OR "Ovarian Neoplasm"[tiab] OR "Ovary Cancer"[tiab] OR "Ovary Cancers"[tiab] OR "Ovarian Cysts"[tiab] OR "Ovarian Cyst"[tiab] 7131 2. (("Ovarian Neoplasms"[Mesh]) OR "Ovarian Cysts"[Mesh]) OR "Ovary/pathology"[Mesh] 84370 3. "Ovarian tumor"[tiab] OR "Ovary tumor"[tiab] 4398 4. 1 OR 2 OR 3 87313 5. ("Diffusion Magnetic Resonance Imaging"[Mesh:NoExp]) OR "Echo-Planar Imaging"[Mesh] 12956 6. "Diffusion Magnetic Resonance Imaging"[tiab] OR "Diffusion MRI"[tiab] OR "Diffusion MRIs"[tiab] OR "Diffusion Weighted MRI"[tiab] OR "Diffusion-weighted MRI"[tiab] OR "Diffusion Weighted MRIs"[tiab] OR "Diffusion-weighted MRIs"[tiab] OR "Diffusion Magnetic Resonance Imaging"[tiab] OR "Diffusion Weighted Magnetic Resonance Imaging"[tiab] OR "Diffusion-weighted Magnetic Resonance Imaging"[tiab] OR "Echoplanar Imaging"[tiab] OR "Echoplanar Imagings"[tiab] OR "Echo Planar Imaging"[tiab] OR "Echo-Planar Imagings"[tiab] OR "Echoplanar Magnetic Resonance Imaging"[tiab] OR "Echo-Planar Magnetic Resonance Imaging"[tiab] OR "Echo Planar Magnetic Resonance Imaging"[tiab] 6942 7. (Diffusion[Mesh] OR Diffusion[tiab]) AND ("Magnetic Resonance Imaging"[Mesh] OR MRI[tiab] OR "Magnetic Resonance Imaging"[tiab] OR MRIs[tiab]) 19752 8. 5 OR 6 OR 7 27922 9. 3 AND 8 86 |
| EMBASE | 1. 'Ovarian tumor':ab,ti OR 'Ovary tumor':ab,ti OR 'Ovarian tumors':ab,ti OR 'Ovary tumors':ab,ti OR 'Ovarian Neoplasms':ab,ti OR 'Ovarian Neoplasm':ab,ti OR 'Ovary Cancer':ab,ti OR 'Ovary Cancers':ab,ti OR 'Ovarian Cysts':ab,ti OR 'Ovarian Cyst':ab,ti 18855 2. 'ovary tumor'/exp OR 'ovary cyst'/exp 134130 3. 1 OR 2 136354 4. 'echo planar imaging'/exp OR 'echo planar imaging' OR 'diffusion weighted imaging'/exp OR 'diffusion weighted imaging' 26923 5. 'Diffusion Magnetic Resonance Imaging':ab,ti OR 'Diffusion MRI':ab,ti OR 'Diffusion MRIs':ab,ti OR 'Diffusion Weighted MRI':ab,ti OR 'Diffusion-weighted MRI':ab,ti OR 'Diffusion Weighted MRIs':ab,ti OR 'Diffusion-weighted MRIs':ab,ti OR 'Diffusion Magnetic Resonance Imaging':ab,ti OR 'Diffusion Weighted Magnetic Resonance Imaging':ab,ti OR 'Diffusion-weighted Magnetic Resonance Imaging':ab,ti OR 'Echoplanar Imaging':ab,ti OR 'Echoplanar Imagings':ab,ti OR 'Echo Planar Imaging':ab,ti OR 'Echo-Planar Imagings':ab,ti OR 'Echoplanar Magnetic Resonance Imaging':ab,ti OR 'Echo-Planar Magnetic Resonance Imaging':ab,ti OR 'Echo Planar Magnetic Resonance Imaging':ab,ti 8401 6. diffusion:ab,ti OR 'diffusion'/de OR 'diffusion coefficient'/exp 174761 7. 'nuclear magnetic resonance imaging'/exp 605133 8. 7 AND 6 34347 9. 4 OR 5 OR 9 45290 10. 3 AND 235 |
| The Cochrane Central Register of Controlled Trials (CENTRAL) | 1. "Ovarian Neoplasms" or "Ovarian Neoplasm" or "Ovary Cancer" or "Ovary Cancers" or "Ovarian Cysts" or "Ovarian Cyst" or "Ovarian tumor" or "Ovary tumor":ti,ab,kw (Word variations have been searched) 2122 2. MeSH descriptor: [Ovarian Neoplasms] explode all trees 1428 3. MeSH descriptor: [Ovarian Cysts] explode all trees 967 4. MeSH descriptor: [Ovary] explode all trees and with qualifier(s): [Pathology - PA] 70 5. 1-4/OR 3041 6. MeSH descriptor: [Diffusion Magnetic Resonance Imaging] this term only 201 7. MeSH descriptor: [Echo-Planar Imaging] explode all trees 74 8. "Diffusion Magnetic Resonance Imaging" or "Diffusion MRI" or "Diffusion MRIs" or "Diffusion Weighted MRI" or "Diffusion-weighted MRI" or "Diffusion Weighted MRIs" or "Diffusion-weighted MRIs" or "Diffusion Magnetic Resonance Imaging" or "Diffusion Weighted Magnetic Resonance Imaging" or "Diffusion-weighted Magnetic Resonance Imaging" or "Echoplanar Imaging" or "Echoplanar Imagings" or "Echo Planar Imaging" or "Echo-Planar Imagings" or "Echoplanar Magnetic Resonance Imaging" or "Echo-Planar Magnetic Resonance Imaging" or "Echo Planar Magnetic Resonance Imaging":ti,ab,kw (Word variations have been searched) 418 9. 6-8/OR 418 10. MeSH descriptor: [Diffusion] explode all trees 131 11. Diffusion:ti,ab,kw (Word variations have been searched) 1884 12. MeSH descriptor: [Magnetic Resonance Imaging] explode all trees 5854 13. MRI or "Magnetic Resonance Imaging" or MRIs:ti,ab,kw (Word variations have been searched) 10812 14. #10 or #11 1884 15. #12 or #13 11125 16. #14 and #15 535 17. #9 or #16 642 18. #5 and #17 2 |
