# Supplementary material for: The Value of Diffusion-Weighted Imaging in the Differential Diagnosis of Ovarian Lesions: A Meta-Analysis
Source: PLoS One. 2016 Feb 23;11(2):e0149465. doi: 10.1371/journal.pone.0149465 (PMC4764370; doi:10.1371/journal.pone.0149465)
Supplement: S2 Table — (DOC) [file pone.0149465.s005.doc]

| Study | **RISK OF BIAS** | | | | **APPLICABILITY CONCERNS** | | |
| --- | --- | --- | --- | --- | --- | --- | --- |
|  | PATIENT SLELCTION | INDEX TEST | REFERENCE STANDARD | FLOW AND TIMING | PATIENT SELECTION | INDEX TEST | REFERENCE STANDARD |
| 1 | 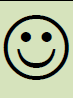 | 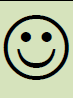 | 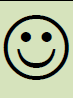 | 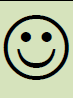 | 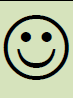 | 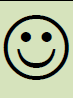 | 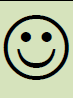 |
| 2 | 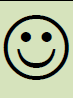 | 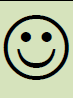 | 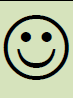 | 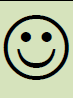 | 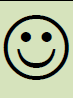 | 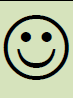 | 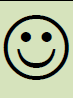 |
| 3 | 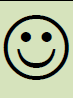 | 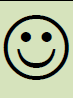 | 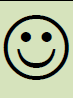 | 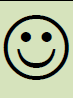 | 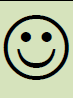 | 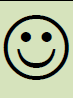 | 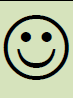 |
| 4 | 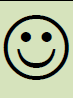 | 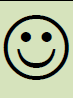 | 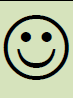 | 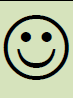 | 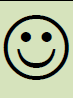 | 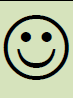 | 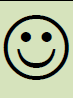 |
| 5 | 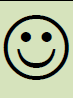 | 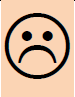 | 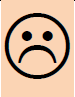 | 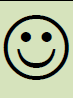 | 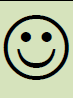 | 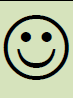 | 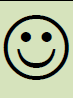 |
| 6 | 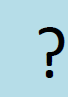 | 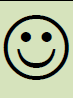 | 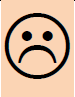 | 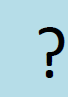 | 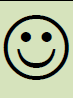 | 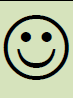 | 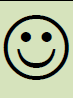 |
| 7 | 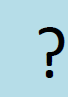 | 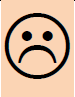 | 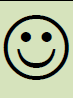 | 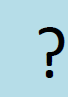 | 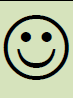 | 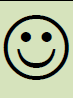 | 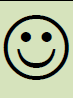 |
| 8 | 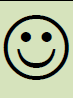 | 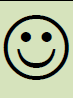 | 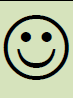 | 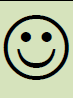 | 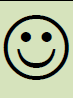 | 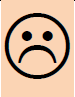 | 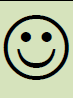 |
| 9 | 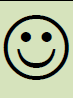 | 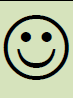 | 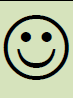 | 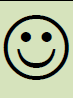 | 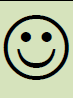 | 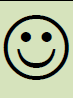 | 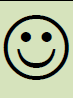 |
| 10 | 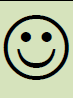 | 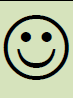 | 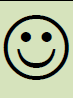 | 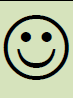 | 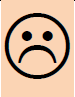 | 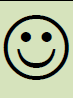 | 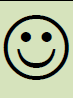 |
| 11 | 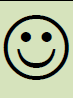 | 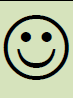 | 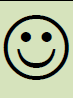 | 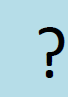 | 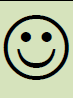 | 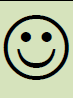 | 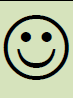 |
| 12 | 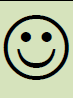 | 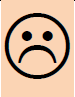 | 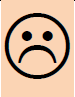 | 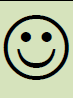 | 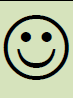 | 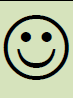 | 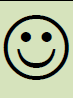 |
| 13 | 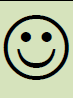 | 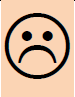 | 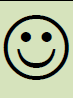 | 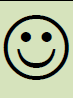 | 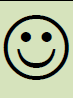 | 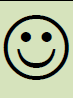 | 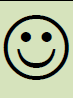 |
| 14 | 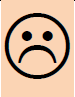 | 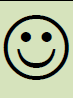 | 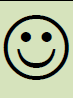 | 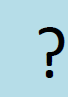 | 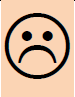 | 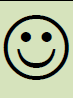 | 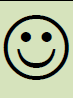 |
| 15 | 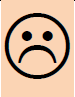 | 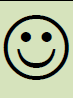 |  |  |  |  |  |
| 16 |  |  |  |  |  |  |  |
| 17 |  |  |  |  |  |  |  |
| 18 |  |  |  |  |  |  |  |
| 19 |  |  |  |  |  |  |  |
| 20 |  |  |  |  |  |  |  |
| 21 |  |  |  |  |  |  |  |

LOW RISK HIGH RISK UNCLEAR RISK
